# Acceptability and feasibility of a mobile health application for blood pressure monitoring in rural Uganda

**DOI:** 10.1093/jamiaopen/ooaa068

**Published:** 2021-01-08

**Authors:** Beatrice Mugabirwe, Tabor Flickinger, Lauren Cox, Pius Ariho, Rebecca Dillingham, Samson Okello

**Affiliations:** 1 Faculty of Computing and Informatics, Mbarara University of Science and Technology, Mbarara, Uganda; 2 University of Virginia School of Medicine, University of Virginia, Charlottesville, Virginia, USA; 3 Department of Internal Medicine, Mbarara University of Science and Technology, Mbarara, Uganda; 4 Lown Scholars Program, Department of Global Health and Population, Harvard T.H. Chan School of Public Health, Boston, Massachusetts, USA

**Keywords:** hypertension, PositiveLinks, mHealth, feasibility, acceptability, self-monitoring, social support, blood pressure control, medication adherence

## Abstract

**Background:**

Mobile technologies to improve blood pressure control in resource-limited settings are needed. We adapted and evaluated the acceptability and feasibility of PositiveLinks, a mobile phone application for self-monitoring, social support, and engagement in care for people living with HIV, among patients with hypertension in rural Uganda.

**Methods:**

We enrolled adults on treatment for hypertension at Mbarara Regional Referral Hospital and Mbarara Municipal health center IV, southwestern Uganda. We provided and educated all participants on the use of PositiveLinks application and automated blood pressure monitors. We administered a baseline questionnaire and performed in-depth interviews 30 days later to explore acceptability, feasibility, medication adherence, social support, and blood pressure control.

**Results:**

A total of 37 participants completed the interviews, mean age of 58 years (SD 10.8) and 28 (75.7%) were female. All participants embraced the PositiveLinks mobile app and were enthusiastic about self-monitoring of blood pressure, 35 (94.6%) experienced peer to peer support. Among the 35 participants non-adherent to medications at baseline, 31 had improved medication adherence. All except 1 of the 31(83.8%) who had uncontrolled blood pressure at baseline, had self-reported controlled blood pressure after 30 days of use of PositiveLinks.

**Conclusion:**

Patients with hypertension in rural Uganda embraced the PositiveLinks mobile application and had improved medication adherence, social support, and blood pressure control. Further assessment of cost-effectiveness of the application in blood pressure control in resource-limited settings will be pursued in future studies.

**LAY SUMMARY OF THE STUDY**

This study evaluated the acceptability and feasibility of the PositiveLinks application, a mobile health technology, among patients with hypertension attending 2 public health facilities in Mbarara, southwestern Uganda. Participants were trained and educated on how to use the application to capture self-reports of medication adherence, mood, social support, and blood pressure. After a month of follow-up, participants showed good acceptability of PositiveLinks and were using the application with ease. This was associated with improvements in self-reported adherence to medication and blood pressure control. Of note, participants expressed access to affordable blood pressure machines and internet as barriers. Overall, PositiveLinks is an easy to use mobile health application associated with improved social support, medication adherence, and blood pressure control among adults with hypertension in a low-resource setting. This was an available solution through a research partnership that required modest modification. Further assessment of cost-effectiveness of PositiveLinks application in blood pressure control in resource-limited settings will be pursued in future studies.

## INTRODUCTION

In Africa, the estimated number of people with hypertension has increased steadily from 54.6 million in 1990 to 130.2 million in 2010, and it is projected to rise to 216.8 million by 2030.^1^ Hypertension is widespread in sub-Saharan Africa (SSA), with East African countries experiencing the highest prevalence in the world.^2^^,^^3^ The high burden of hypertension has severe consequences including increased cardiovascular diseases (stroke, myocardial infarction, and hypertensive heart diseases) morbidity and mortality.^4^

Compared to North African countries, countries in SSA have low levels of control of hypertension especially in rural areas.^5^ Poor blood pressure control in SSA has been attributed to poor health infrastructure and compliance to treatment, with poverty being the underlying cause.^6^ Lack of personal blood pressure monitoring^7^ is a barrier to blood pressure control. Home-based blood pressure measurements correlate more closely with ambulatory blood pressure monitoring^8^^,^^9^ and predict cardiovascular disease better than the clinic-based blood pressure measurements.^9–12^ Affordable and easy to use alternatives that could reduce unnecessary clinic visits and involve patients in their care through blood pressure self-monitoring, are needed in SSA.

PositiveLinks is a mobile health application designed to facilitate self-monitoring, social support, and engagement in care initially developed for people living with HIV.^13^^,^^14^ PositiveLinks application has been used within the United States and internationally for HIV interventions but its features are potentially applicable to other chronic conditions, such as to enhance remote blood pressure management in settings where access to care impacts on blood pressure control like rural Uganda.^15^ We aimed to adapt PositiveLinks for patients with hypertension and assess its acceptability and feasibility for blood pressure control in rural southwestern Uganda.

## METHODS

### Study setting and participants

This pilot study was a mixed methods cohort study of patients with hypertension, resident and receiving clinical care at Mbarara Regional Referral Hospital and Mbarara Municipal Council Health Center IV, southwestern Uganda. These 2 public health facilities represent the setting where most patients seek clinical care for hypertension and as such the findings may be generalizable to similar public health facilities in SSA. A stratified random sampling was done at baseline for potential participants of the study, the sample size was 206. For PositiveLinks, we used consecutive sampling to enroll adults aged 30 years or greater, who owned an android mobile phone, residents of Mbarara town (5 km), who should have taken antihypertensive medication for at least 4 months, and receiving clinical care for hypertension at the outpatients’ clinics in either of the 2 facilities. Pregnant women were excluded as hypertensive disorders of pregnancy were out of the scope of this study. As a pilot study to demonstrate the acceptability and feasibility of PositiveLinks, we did not carry out a sample size calculation but targeted at least 30 participants.

### Ethical review

Ethical approval for this study was obtained from the Institutional Review Committee of Mbarara University of Science and Technology and the Uganda National Council for Science and Technology. Participants provided signed informed consent before study participation.

### Study procedure

A questionnaire was administered at baseline and an in-depth interview was conducted on each participant after 30 days of use of PositiveLinks and blood pressure machine. The 30-day period was used since it coincides with the scheduled clinical visits when medication would be replenished.

### PositiveLinks application

**Figure 1.**
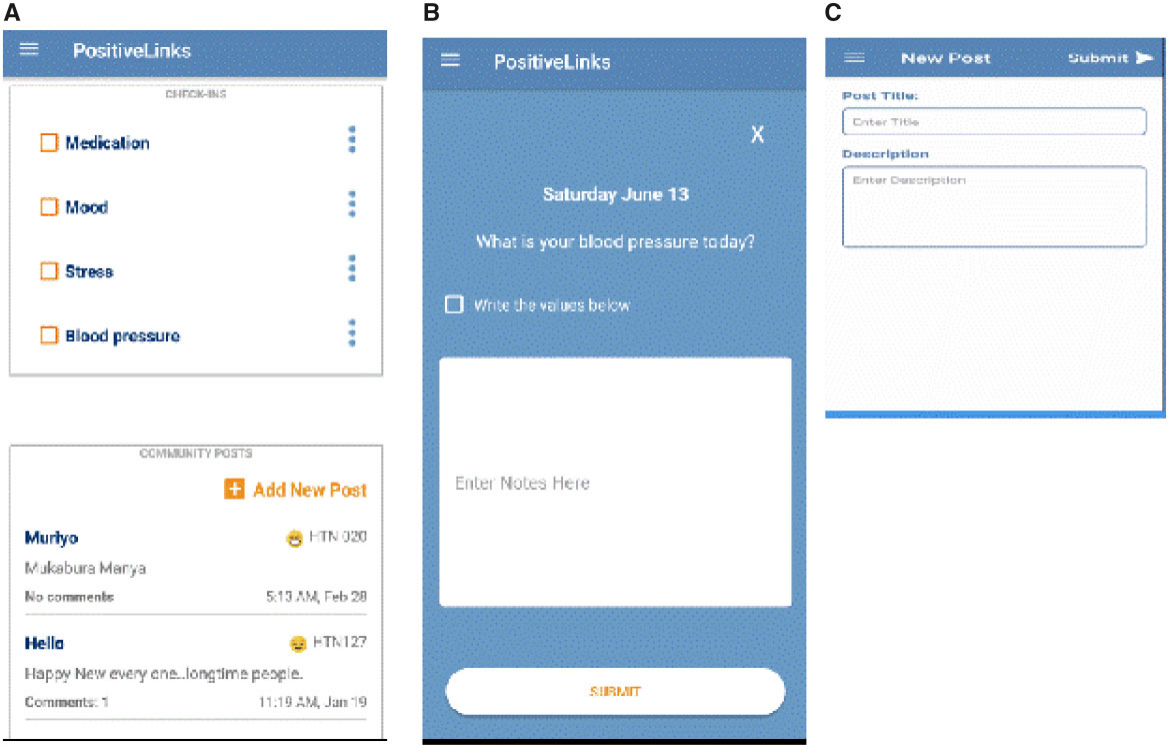
Showing the check-ins of the application and community board messages (A), blood pressure check-in only (B), and input screen for a new community message (C).

The study team customized the questions in the PositiveLinks application for hypertension from the HIV related questions for which this application was built.^13^ Briefly, prompts and questions were adjusted to be understandable by laypeople with minimum explanation in English language. PositiveLinks application is a mobile health intervention designed to facilitate self-monitoring, social support, and engagement in care initially developed for people living with HIV.^13^ This mobile application was customized to suit hypertension patients from low resource settings. The source code was originally provided by the PositiveLinks Team from the University of Virginia, Charlottesville, USA. The application initially contained HIV-related questions which were changed to suit hypertensive patients. After multiple iterations, the final version was pretested among the study team members based in Mbarara for cultural context. PositiveLinks application contained check-Ins (Figure 1) which were used to capture data on **self-monitoring** and these included medication adherence (All, some and none), stress (Low, medium and high), mood (Very happy, happy, OK, unhappy and very unhappy) and blood pressure readings (systolic and diastolic blood pressure). The application was also designed to provide s**ocial support** through a virtual community message board (Figure 1) allowing anonymous messaging among participants. Participants included in the study received the PositiveLinks application, this was installed on their Android phones. All participants were trained individually on how to access the application and its different functionalities such as the virtual community message board used for communication among participants, entry check-ins for self-reported mood, stress, medication adherence, and blood pressure readings. The application was password protected. All entries were captured and stored in a secure online database. All participants were provided with prepaid internet bundles to ensure continuous internet connectivity using the app. Participants were remotely monitored by the research coordinator through the real-time database and phone calls.

Enrolled participants were trained on the standard procedure for blood pressure measurement using automatic sphygmomanometers (Omron^®^ BP710N 3 series, Omron Healthcare Inc., Bannockburn, USA) with appropriate cuff sizes as per arm circumference i.e., small (<21 cm), normal (22–32 cm), and large cuffs (35–44 cm).^16^ Participants were instructed to do daily blood pressure measurements, immediately upon waking in the morning and as the last thing before bed in the night. These measurements were then entered into the PositiveLinks application.

On the study exit, one investigator proficient in English and the local language (Runyankole) carried out individual in-depth interviews to explore detailed participants’ perspectives, understanding, and experiences using the application for hypertension management. The audio-recorded interviews were translated to English to ensure transcription by other investigators who also did reviews for quality, clarity, and detailed information.

### Data analysis

**Figure 2.**
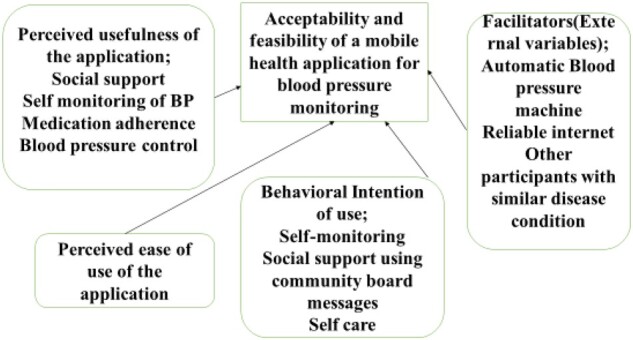
Showing the organization of qualitative data on acceptability and feasibility following the technology acceptance model: blood pressure control, social support and medication adherence using PositiveLinks® application.

We used Dedoose software program to analyze the data.^17^ We reviewed content relevant to acceptability and feasibility drawn from the Technology Acceptance Model (TAM) theory,^18–20^ then sorted the codes from the data, and reviewed to develop descriptive categories in line with the domains of the TAM (perceived usefulness, perceived ease of use, behavioral intention of use and external variables) as indicated in Figure 2.^18–20^ We iteratively developed the coding strategy until thematic saturation and inter-coder reliability were achieved. Coding discrepancies were resolved by consensus. Lastly, illustrative quotes from participants were selected from the coded data.

## RESULTS

### Participants’ social demographic characteristics

Of the 206 patients with hypertension sampled between September 2019 and January 2020, 38 participants were enrolled for the pilot study. We excluded 99 non-residents of Mbarara, 138 had analog/button phones, 18 had <4 months of hypertension diagnosis, and 3 did not own a mobile phone. Among the 38 enrolled, one participant withdrew consent during follow-up and was excluded from analysis. Of the 37 participants who completed the 30-day interviews, the mean age was 58 years (SD 10.8) years, 28 (75.7%) were female, and median duration of hypertension diagnosis was 5 years (interquartile range 3–5 years). All participants actively used the application until study exit (Table 1).

**Table 1.** Participants’ social demographic characteristics

Characteristics		Statistics
Participants included in the intervention		38 (100%)
Participants who completed the study, *n* (%)		37 (97.4%)
Gender		
Male		9 (24.3%)
Female		28 (75.7%)
Over all mean (SD) age		58 (10.846)
Median hypertension duration		5 (3–5 years)

Evaluation of acceptability and feasibility of the application were carried out drawing from the domains of TAM^18–20^ associated with the components of the study which are social support, blood pressure control, and medication adherence of hypertension participants as indicated in Figure 2.

### Perceived usefulness

*Social support*: 35 (94.6%) experienced peer social support through posts and replies from the virtual community board messages;

“*It’s like a support group, when you are down, you go there, people try to post and you don’t feel down again. If you are having a challenge with your health, with your diet, you go there and there’s always somebody there posting so it’s like a family member whom you have not met but is actually helping, so we have each other’s back, you understand, we help each other. If somebody has a question, there’s always somebody to answer and assist, so it’s really fantastic.” (****Female, 75 years old****)*

*Improves self-monitoring of BP*: Participants found using the mobile health application as a real-time and convenient aid to self-monitoring of their daily blood pressure;

*“Well, it has helped me in monitoring my high blood pressure and knowing how I am daily. I see my blood pressure is better compared to when I used to go the hospital after a longtime minus monitoring.” (****Male, 76 years old****)*

*Improves self-reported medication adherence*: Among the 35 participants who reported missing taking their medications at baseline, 31 reported taking all medication as prescribed at the end of the study. The participants attributed this to reminders that helped them remember to take their medication.

“*we are able to enter our blood pressure readings and monitor it and then it reminds to take our medication because we enter that we didn’t take our medication, it will warn you that it’s important to take your medication and you can also communicate with a doctor in case so I think to me it’s perfect.” (****Female, 52 years old)***

*Blood pressure control*: Among the 31(83.8%) who uncontrolled blood pressure at baseline, all except 1 had had controlled blood pressure after 30 days. Some participants attributed the stability in their bp to use of the app;

*“Yes I have seen the change, like I said earlier, my blood pressure has stabilized in good figures because before I started using the application, whenever I would go to the hospital, my blood pressure would be high but now it has stabilized.” (****Male, 76 years old)***

### Feasibility of the PositiveLinks application

*Ease of use*: Participants found the application easy to use during self-management;

*“No challenges whatsoever, it’s very easy to use and I know that when my blood pressure is too high, I have to take my medication and not miss it, if the blood pressure stays high even after taking my medication, I have to contact my doctor so there’s really no challenge because it’s very easy to use.” (****Male, 76 years old)***

*Behavioral intention of use*: Participants stated that they became accustomed to self-monitoring through the use of the application, which helped them in managing and controlling their hypertension:

*“It helped us to be aware of our hypertension, that it’s there yes and also how to manage it, actually we have been forced in a good and cooperative way to manage our blood pressure, because now you observe we have hypertension but knowing you have hypertension, you are forced to take care of it and I have a feeling that it’s going to be a continuous process for us because having experienced this nobody would want to divert back to that period when we were careless about our hypertension.” (****Female, 52 years old)***

Community board messages using the application among participants gave lifestyle tips on diet and exercise to control their hypertension;

*“It added, like you know, people talk about exercising and walking so we get their other things other than the medication, because nobody there can advise you on what medication to take apart from the doctor but other additional things like dietary, exercise, taking things easy and having enough sleep, all these things, you know people were reminding us on the new fruits to have you know, how to use medication to work better.” (****Female, 52 years old)***

Usage of the app enabled participants to know their blood pressure status and how best to care for themselves;

*“Knowing one’s health is very important because now I would get to monitor myself and know how I am feeling, whenever I would take my measurements, knowing that its hypertension may be making me weak and I find it’s normal then I would know its other ailments that are disturbing me and I would know what to treat. This made me very happy.” (****Female, 57 years old)***

### Facilitators

*Automatic blood pressure machine*: Participants used the bp machines for daily measurements while using the application;

*“The advantage was leaving me with the application and machine for a month, because of this I don’t have to borrow the machine from any one because I already have one or go to the hospital! I have this machine here in my house and I can take my measurements anytime of the day.” (****Female, 46 years old)***

*Internet*: Reliable internet access was readily provided to the participants;

*“I had enough [internet] data and thus nothing could hinder me from seeing the messages or sharing with the members.” (****Female, 62 years old)***

*Other participants with similar disease conditions*: Other participants with similar disease conditions made the use of the application easier;

*“It was really helpful, it was easy to use, and not difficult and you don’t waste time, the network was cooperative and so it was so easy to use and aah you could also communicate on the platform with other people with a local community, communicating your experiences with each other, comforting each other, yea it was a very good thing, it helped me with my high blood pressure management.” (****Female, 52 years old)***

### Challenges

However, participants reported challenges both internal and external, which hindered the use of the PositiveLinks application.

*Forgetfulness*: Participants stated difficulty in using the application sometimes due to forgetfulness;

*“Well the problem is, I don’t know how to use the application well as I should, sometimes when I forget some things I call my son who helps me to enter my information when such issues arise, so I use a few features in the application.” (****Female, 46 years old)***

*Fear of knowing daily blood pressure measurements*: Some participants felt scared to do self-monitoring using the application daily, for fear of knowing that their blood pressure might be high;

“*Well when you gave me these things I felt happy because my blood pressure usually goes high, sometimes it’s so very bad, but whenever I would take my measurements and its extremely high I would get scared so at some point I stopped monitoring myself because my bp was not going down and I was scared and frustrated, then sometimes I would feel bad.” (****Female, 59 years old)***

*High cost of antihypertensive medication or cuffs*: Difficulty in affording medication or blood pressure cuffs and trouble accessing medical care were challenges for some participants;

*“I request that you try to help us access medication or increase the machines in the hospital, because sometimes we don’t have medication which is a problem, sometimes we don’t even have money.” (****Female, 70 years old)***

### Improvements

*Need for more understanding of application and disease conditions*: Some participants stressed the need for extra training on how to exactly use the application in the management of hypertension;

*“Well I would love much more awareness on these items in as much as I was using the application and the machine, I needed much more information, I suggest that training should involve many things and more information and you give it enough time like an entire day per person so that we get to understand much more about the items and what high blood pressure truly is. We have it but we are not well knowledgeable about it that much.” (****Female, 75 years old)***

*Healthcare providers’ communication*: Participants suggested that health care providers try to communicate and support participants as a form of encouragement using the intervention;

*“I don’t know because we have really helped each other, I think it may be on your part, we want you to encourage participants for real-time support groups because it really helps not only hypertension but can help even groups with other diseases, most people actually fail in managing themselves because they don’t have such support group but if you start real-time support groups it would help much better, here most people reply at their own time and pace.” (****Female, 52 years old)***

## DISCUSSION

Drawing from TAM theory,^18–20^ we found that PositiveLinks application supplemented with an affordable blood pressure cuff and reliable internet is acceptable and feasible for supporting hypertensive patients to control blood pressure, receive social support, and improve medication adherence in rural southwestern Uganda. These findings are reassuring in the Ugandan context where facilitators as such phone coverage is 81% while broadband coverage is 37%.^21^ Of note, phone ownership cuts across education level, age, economic status, gender and is independent of residence in urban or rural areas.^22^ Taken together, increasing access to phones and the internet enables the use of health intervention and disease prevention tools (mHealth or eHealth tools) that reach patients beyond the geographical clinical settings as such augmenting the overstretched health system. Our findings are consistent with those in other settings that showed that mobile applications are generally acceptable by users and effective in blood pressure management.^23^^,^^24^

Acceptability and feasibility of PositiveLinks application were promoted by the perceived usefulness, ease of use, and participants’ satisfaction. Participants pointed out the ability of the PositiveLinks application to establish social networks using the virtual private network (community board messages). The social support improved the relationship among participants, who enjoyed messaging each other, improving their mood, and reducing their stress. Peer to peer support and self-care can help to prevent negative psychological effects^25^^,^^26^ among hypertensive patients using mobile health applications. This effect of improving social support by the ability to communicate with others shows that well-designed mhealth tools that allow messaging have desirable effects needed for chronic disease management.^27–29^

PositiveLinks application showed improvement in health outcomes among patients living with hypertension as evidenced by self-reported adherence to antihypertensive medications and improved blood pressure control. These clinically important findings are in keeping with others that showed that mobile health applications play a major role in treatment and improving blood pressure control and management.^27^^,^^30^^,^^31^ However, these studies did not have educational messages for hypertension management.^25^^,^^28^^,^^29^

Our findings should be appraised within the context of our study limitations. First, this was a 2-center pilot study with a small sample. Second, the application was customized in English only, which limits the generalizability of our results to participants who cannot read English. Third, there was potential for selection bias if participants who chose to enroll in the study differ from others in the population. There were more women than men in the study, which may be due to women being better health seekers than men. Fourth, residing within a 5 km radius from the clinics as an inclusion criterion could have been a proxy for both mobile phone/internet access and use in this group. Fifth, smart phone ownership as our inclusion criterion was a limiting factor to this study considering majority of the potential participants having analogue phones, thus directly limiting the feasibility uptake. Thus, attempts to generalize these results to other populations should be made with caution.

However, the current study provides a profile of mobile phone use to inform future mhealth interventions in similar settings. In addition, this study has potential health policy implications in emphasizing the importance of reliable internet access in the management of medical conditions. Efforts to improve internet access in low-resource settings may help reduce health disparities by enabling more equitable participation in mhealth interventions.^32^

The strengths of this study include: (1) our sample consisted of adults receiving clinical care for hypertension at one tertiary and one low level public health facilities from peri-urban and rural settings where both mobile phone/internet access and use are expected to be comparable to other parts of the country as such the findings are generalizable to Uganda and other low-income countries; (2) We used the TAM, a standard model theory, to access the technology acceptance of the application.

In conclusion, we found that PositiveLinks application, a low cost, low maintenance, and easy to use mobile health application, can be rolled out to improve social support, medication adherence, and blood pressure control among adult hypertensive patients in a low-resource setting. This was an available solution through a research partnership that required modest modification. Further assessment of cost-effectiveness of PositiveLinks application in blood pressure control in resource-limited settings will be pursued in future studies.

## FUNDING

This work was supported by the center for Global Health, grant number (None) at University of Virginia School of Medicine, Charlottesville, VA, USA.

## AUTHOR CONTRIBUTIONS

All authors duly contributed to this manuscript and they all certify that they sufficiently participated and are responsible for this work. All authors confirm that this work is not under consideration elsewhere. All authors meet authorship criteria and none have disclosures or conflicts of interest relating to the study. These are the contributions from all authors as follows: Ms. Beatrice Mugabirwe took part in writing the concept and design of the study, data collection, analysis and interpretation of data, drafting of the manuscript, revision and critiquing of the manuscript, and approval of the final version of the manuscript. Dr. Tabor Flickinger took part in analysis and interpretation of data, drafting of the manuscript, revision and critiquing of the manuscript, and approval of the final version of the manuscript. Ms. Lauren Cox took part in analysis and interpretation of data, drafting of the manuscript, revision and critiquing of the manuscript, and approval of the final version of the manuscript. Dr. Pius Ariho took part in analysis and interpretation of data, drafting of the manuscript, revision and critiquing of the manuscript, and approval of the final version of the manuscript. Dr. Rebecca Dillingham took part in writing the concept and design of the study, drafting of the manuscript, revision and critiquing of the manuscript, and approval of the final version of the manuscript. Dr. Samson Okello supervised and was actively involved in writing the concept and design of the study, analysis and interpretation of data, drafting of the manuscript, revision and critiquing of the manuscript, and approval of the final version of the manuscript.
